# Molecular analysis of the microbiota in hard feces from healthy rabbits (*Oryctolagus cuniculus*) medicated with long term oral meloxicam

**DOI:** 10.1186/1746-6148-10-62

**Published:** 2014-03-11

**Authors:** David Eshar, J Scott Weese

**Affiliations:** 1 Department of Clinical Sciences, College of Veterinary Medicine, Kansas State University, Manhattan, Kansas 66506, USA; 2 Department of Pathobiology, Ontario Veterinary College, University of Guelph, Guelph, Ontario N1G 2 W1, Canada

**Keywords:** Microbiome, Rabbits, Gastrointestinal tract, Feces, 16S RNA, Meloxicam, *Oryctolagus cuniculus*

## Abstract

**Background:**

Analgesia is often indicated in rabbits undergoing surgical procedures or suffering from various painful conditions and the most common adverse effects associated with NSAIDs occur in the gastrointestinal tract (GIT). The objective of this study was to determine the potential effect of long-term (21 days) meloxicam administration on the fecal bacterial microbiota in healthy rabbits.

Samples of hard feces were collected from six rabbits treated with meloxicam (1 mg/kg orally once every 24 h) on days 0,6,14 and 21. Next generation sequencing of V4 16S rRNA gene products was performed.

**Results:**

A total of 2589912 V4 rRNA gene sequences passed all quality control filters. Firmicutes predominated (82.0 ± 6.2%). Sixteen other phyla were also identified but other than Verrucomicrobia (4.4 ± 4.9%), all accounted for less than 1% of the identified sequences. Within Firmicutes, Clostridia was the dominant class, accounting for 76% of operational taxon units (OTUs). In general, there were only few differences observed between time points and different rabbits at the phylum level. A significant change was observed in the relative abundance of Proteobacteria over the 4 time points (*P* = 0.02).

**Conclusions:**

The gastrointestinal tract of rabbits harbors dense and diverse microbiota. Significant alteration of the hard fecal microbiota does not appear to be a considerable adverse effect expected in rabbits treated for 21 days with oral meloxicam at a dose of 1 mg/kg.

## Background

Analgesia is a basic aspect of veterinary medicine, indicated in both laboratory and pet-owned animals undergoing surgical procedures or suffering from various painful conditions [1]. One of the most common classes of drugs used by veterinarians for analgesia is non-steroidal anti-inflammatory drugs (NSAIDs) [1]. Nonsteroidal anti-inflammatory drugs reduce inflammation by inhibiting the action of Cyclooxygenase (COX) enzymes, which convert arachidonic acid into prostanoids and analgesia associated with NSAIDs is mainly due to inhibition of COX-2 [2]. The most common adverse effects associated with NSAIDs occur in the gastrointestinal tract (GIT), and to a lesser degree, impairment of renal blood flow [3,4]. Reported NSAIDs-related GIT pathologies include perforation, ulceration, bleeding and local irritation [3,4]. Because prostaglandins also play a role in the rabbit’s GIT motility [5], NSAIDs have the potential to also impair GIT function. To date, several studies have been performed to determine pharmacokinetics and safety of meloxicam in rabbits but none had looked into the potential drug’s effect on GIT microbiota [6-8].

The fecal or intestinal microbiota of domestic rabbits has been extensively studied through culture dependent methods, as well as earlier generation culture-independent methods, with a focus on the cecal microbiota [9-13]. However, limitations of these methods for understanding the overall microbial composition are well known. In particular, culture-dependent studies are only able to provide a very superficial (and sometimes misleading) assessment of this complex polymicrobial environment [14-16]. While earlier studies have provided some important information, the ability of next generation sequencing based studies such as this to evaluate millions of bacterial sequences from diverse (and often unculturable) microbial populations is now possible.

This study explores the stability over time of bacterial communities in hard feces in healthy rabbits medicated with oral meloxicam, using high throughput sequencing of the V4 region of the 16S rRNA gene.

## Methods

### Study population

Ethical approval was obtained from the Institutional Animal Care and Use Committee of Kansas State University. Rabbits enrolled in this study were part of a broader study evaluating long term (29 days) administration of oral meloxicam. Six clinically normal 3-month-old intact female New Zealand White rabbits (body weight range, 2.52 to 2.71 kg) were included in this study. The rabbits were obtained from a commercial source and were specific pathogen (*Pasteurella* sp.) free. The rabbits were housed individually at the research facilities of the Kansas State University College of Veterinary Medicine at a constant temperature (21°C) and humidity (60%), and were exposed to cycles of 16 hours of light and 8 hours of dark per day. Rabbits were fed free choice of timothy hay^a^, timothy-based pelleted diet^b^ and water was available ad libitum. Rabbits were acclimated to the facility for 5 days after their arrival and were habituated to handling prior to initiation of the study. Immediately prior to the start of the study, each rabbit was determined to be behaviorally normal and healthy based on a thorough physical examination and clinical pathology testing (complete blood count [CBC], serum biochemistry profile and urinalysis), with results compared to published reference ranges [17]. Physical examination, weight and blood testing were repeated weekly in all rabbits enrolled in the study. Fecal production was subjectively monitored for shape, color and consistency, and objectively by weight and fecal pellet size. At the end of the study, all rabbits were humanely euthanatized using pentobarbital^c^ (100 mg/kg Intravenous), and necropsy was performed within 15 minutes of euthanasia.

### Experimental design and sample collection

Meloxicam^d^ (1 mg/kg) was orally administered daily for 29 days to each rabbit via a 3-mL syringe. The rabbits’ behaviour, attitude, mentation, level of activity, eating and drinking, and defecation were subjectively assessed daily by the researchers. Hard fecal samples were collected prior to treatment (day 0) and on days 6, 14 and 21 from the start of the meloxicam treatment. At each time point morning (08:00), 10 fecal pellets from a fresh elimination were placed into an individual collection bag. Therefore, four fecal samples were collected from each rabbit during the study period for determination of fecal microbiota. Each sample (10 fecal pellets) was weighed using a digital gram scale and each fecal pellet was measured using hand calipers. All bagged samples were stored at −70°C until analysis.

### DNA extraction and quality control

DNA extraction was performed using a commercial kit^e^ following the manufacturer’s “stool DNA protocol for pathogen detection”, which includes an initial bead beating step and proteinase K treatment. DNA quantity and quality were accessed by spectrophotometry using the NanoDrop^f^.

### 16 s rRNA gene amplification and sequencing

PCR amplification of the V4 region of the 16S rRNA gene was designed based on Klindworth et al. [18] using the primers forward S-D-Bact-0564-a-S-15 (5′-AYTGGGYDTAAAGNG-3′) and reverse S-D-Bact-0785-b-A-18 (5′-TACNVGGGTATCTAATCC-3′). The forward and reverse primers were designed containing an overlapping region of the forward and reverse Illumina sequencing primers (TCGTCGGCAGCGTCAGATGTGTATAAGAGACAG and GTCTCGTGGGCTCGGAGATGTGTATAAGAGACAG, respectively) in order to anneal them to primers containing the Illumina adaptors plus the 8 bp identifier indices (forward: AATGATACGGCGACCACCGAGATCTACAC-index-TCGTCGGCAGCGTC; reverse: CAAGCAGAAGACGGCATACGAGAT-index-GTCTCGTGGGCTCGG). For a final volume of 50 μL, 2 μL of each DNA sample were added to a solution containing 18.7 μL of water, 25 μL of ReadMis^g^, 1.3 μL of BSA^h^, 0.5 μL of each 16S primer (1000 pmol/μL) and 1 μL of each Illumina primer (1000 pmol/μL). The mixture was subjected to the following PCR conditions, 3 minutes at 94˚C for denaturing, and 35 cycles of 45 seconds at 94˚C for denaturing, 60 seconds at 50˚C for annealing and 90 seconds at 72˚C for elongation followed by a final period of 10 minutes at 72˚C and kept at 4˚C until purification.

PCR products were evaluated by electrophoresis in 2% agarose gel and purified with the Agencourt AMPure XP^i^ by mixing 22 μL of amplicon with 72 μL of AMPure on a 96 well plate. After 5 minutes at room temperature, beads were separated and washed twice with 80% ethanol and eluted in 30 μL of water. After purification samples were quantified by spectrophotometry using the NanoDrop^j^ and normalized to a final concentration of 2 nM. Sequencing of the library pool was performed at the University of Guelph’s Advanced Analysis Centre using an Illumina MiSeq^k^ and 2x250 chemistry.

### Microbiome assessment

The MOTHUR package of algorithms (v1.30) was used for analysis [19]. Paired end reads were aligned. Sequences >275 bp and those containing ambiguous base calls or long runs (>8 bp) of holopolymers were removed, as were sequences that did not align with the correct region. Chimeras were detected using uchime [20] and removed. Sequences from chloroplasts, mitochondria, Archaea and eukaryotes were also removed.

Sequences were binned into OTUs at a 3% (0.03) dissimilarity level, and taxonomy was assigned using the Silva 16S rRNA reference database [21].

Coverage was assessed using Good’s coverage. Population diversity was described using the inverse Simpson’s index. Relative abundances were compared between animals and between timepoints using ANOVA.

Dissimilarity between the two groups was assessed through creation of dendrograms using the Yue & Clayton measure of dissimilarity (a measure of community structure, which considers shared OTUs and their relative abundances) and traditional Jaccard index (a measure of community membership, which considers the number of shared OTUs, not their abundance). Parsimony and unweighted unifrac [22] tests were applied to evaluate the impact of day of treatment on population structure. Principal coordinate analysis (PCoA) was also performed, based on both Jaccard and Yue and Clayton indices. Analysis of molecular variance (AMOVA), a non-parametric evaluation of genetic diversity, was used to evaluate the impact of day.

## Results

Meloxicam was easily administered daily to each rabbit, and all of the rabbits seemed to remain healthy during the study. Subjectively, none of the rabbits had changes in behavior, attitude, mentation, level of activity, amount of food or water consumed, or fecal production. Objectively, all participating rabbits continued to gain weight (final body weight range 2.61-2.91 Kg) when compared to the beginning of the study period. Also, all values in the weekly serum biochemistry analysis were within normal limits both before and during the study. No changes were observed in hard feces’ shape, color and consistency (no diarrhea or ileus). The mean weight of a 10-pellet fecal sample was 6.75 g (range 6–9 g) and mean measured individual fecal pellet size was 6.2 mm (range 5–8 mm). Post-study gross necropsy and histopathologic evaluation of all participating rabbits did not reveal any major abnormalities.

**Figure 1:**
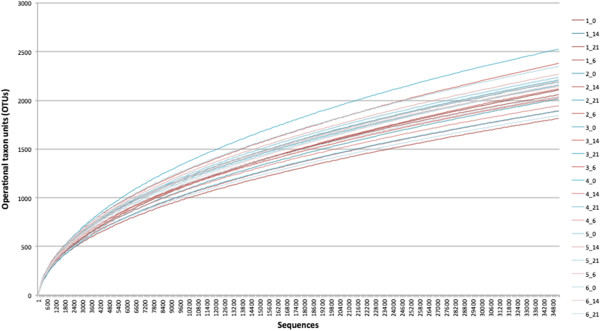
**Rarefaction curve demonstrating the change in number of operational taxon units (0.03 level) with increasing sequence numbers in 6 rabbits over 4 different timepoints.** Legend: rabbit number_sample day.

A total of 3453182 V4 16S RNA gene sequences were obtained; 2589912 of which passed all quality control filters. The number of sequences per sample ranged from 35178 to 250720 (mean 107913, SD 42703). Further analysis was performed on random subsampling of 35178 sequences per sample. Excellent sample coverage was obtained with this subsampled population, as demonstrated by a mean Good’s coverage value of 0.972 (SD 0.002, range 0.967-0.975) and rarefaction curves (Figure 1). The number of OTUs ranged from 1814–2525 (mean 2118, SD 170). There was no impact of rabbit (P = 0.16) or day (P = 0.16) on the number of different OTUs that were identified. The overall mean inverse Simpson’s value was 73.6 (SD 23.6, range 19.5-119), with no impact of day (P = 0.54) or rabbit (P = 0.07).

**Figure 2:**
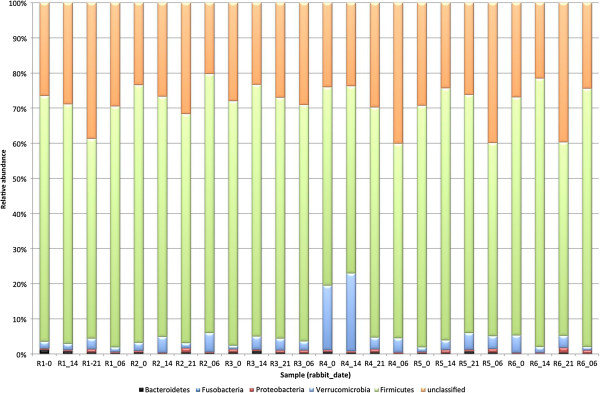
Distribution of the main phyla of the fecal microbiota of 6 healthy adult rabbits (R1-R6) at four different time points (days 0, 6, 14 and 21) after meloxicam treatment.

The microbiota of all rabbits was dominated at the phylum level by Firmicutes, which accounted for 66.6 ± 6.7% (mean ± SD) of sequences (Figure 2). Sixteen other phyla were identified; however, only Verrucomicrobia (4.4 ± 5.1%) and accounted for greater than 1% of sequences. These other phyla consisted of Actinobacteria (0.18 ± 0.13%), Bacteroidetes (0.38 ± 0.26%), Chlamydiae (0.001 ± 0.00%), Cyanobacteria (0.003 ± 0.003), Deinococcus-Thermus (0.0001 ± 0.0005%), Fibrobacteres (0.021 ± 0.014%), Fusobacteria (0.003 ± 0.002%), Lentisphaerae (0.002 ± 0.002%, Plantomycetes (0.0002 ± 0.0008), Proteobacteria (0.57 ± 0.33%), Spirochaetes (0.043 ± 0.24%), Synergistetes (0.005 ± 0.001%), Tenericutes (0.021 ± 0.017%), SR1 (0.0006 ± 0.001%) and TM7 (0.06 ± 0.07%). Sequences that were unclassified at the phylum level accounted for 27.7 ± 5.5% of sequences.

Within Firmicutes, Clostridia was the dominant class, accounting for 76% of OTUs from Firmicutes, with Ruminococcaceae and Lachnospiraceae being the main families (order, Clostridiales), representing 48 and 20% of sequences from Clostridia, respectively. Unclassified Clostridia were common (24%), followed by smaller numbers of Bacilli (2.9%).

Within Verrucomicrobia, Verrucomicrobiaceae was the predominant (98%) family. Within that family, *Akkermansia* and *Persicirhabdus* were the two identified genera, but they only accounted for 9.9 and 0.2% of sequences from Verrucomicrobia, respectively. The remaining members of that family were unclassified at the genus level.

There were few differences between different timepoints and different rabbits at the phylum level. Rabbit 4 had a significantly higher relative abundance of Verrucomicrobia (12%) compared to the others (2.1-3.5%) (P = 0.03). No other differences between rabbits were evident at the phylum level. There was a significant change in the relative abundance of Proteobacteria over the four timepoints (*P* = 0.02), with day 21 having a significant higher relative abundance than the other three time points. Otherwise, no significant differences between timepoints were identified.

**Figure 3:**
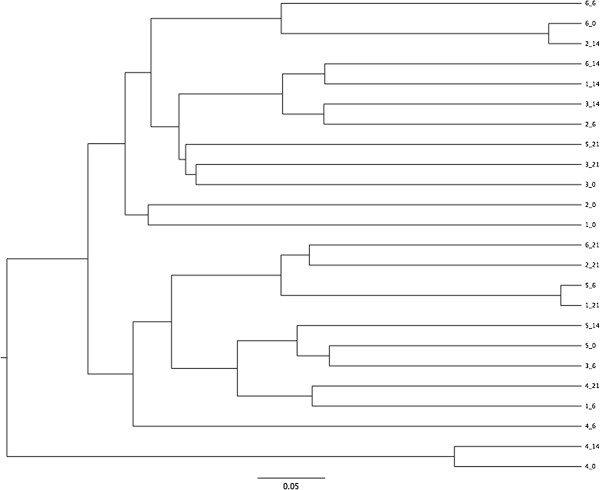
**Dendrogram depicting the Yue & Clayton measure of dissimilarity for six rabbits that underwent meloxicam treatment.** Legend: identifiers indicate Rabbit_sampling day.

**Figure 4:**
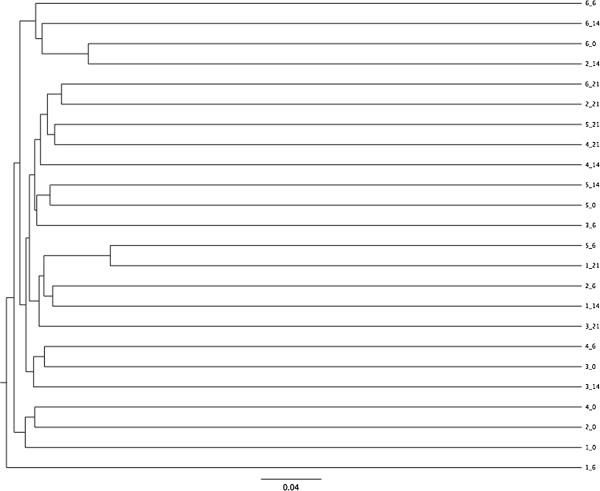
**Dendrogram depicting fecal microbial community membership of six rabbits that underwent meloxicam treatment based on the traditional Jaccard index.** Legend: identifiers indicate Rabbit_sampling day.

Dendrograms based on Jaccard and Yue & Clayton indices are presented in Figures 3 and 4. There were few differences in microbial population structure. No effect of sampling day was evident by unweighted unifrac (Table 1). A significant difference was noted with parsimony test but only between days 0 and 21 and using the Jaccard index.

**Table 1:** Comparison of the microbial populations of six rabbits over four timepoints after treatment with meloxicam

	**Unweighted Unifrac**		**Parsimony**	
	**Jaccard (*P *** **value)**	**Yue and Clayton (*P *** **value)**	**Jaccard (*P *** **value)**	**Yue & Clayton (*P *** **value)**
Day0-Day6	0.48	0.83	0.16	0.56
Day0-Day14	0.71	0.80	0.56	0.57
Day0-Day21	0.17	0.32	0.02	0.58
Day6-Day14	1.00	0.80	0.94	0.57
Day6-Day21	0.73	0.89	0.93	0.93
Day14-Day 21	0.58	0.14	0.53	0.54

**Figure 5:**
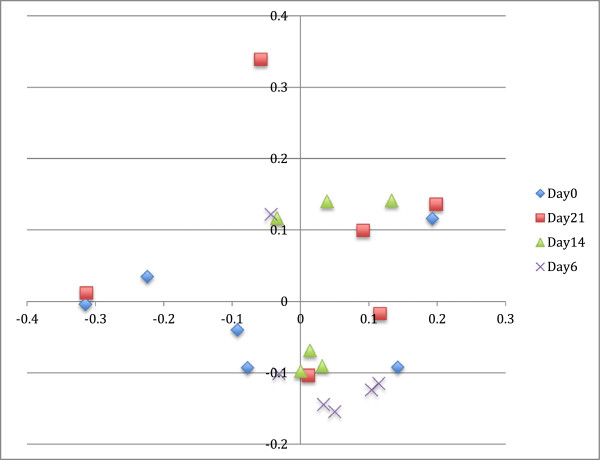
**Three dimensional principle coordinate analysis, highlighted by treatment day.** Legend: 5a:open box-day0; + = day6; closed circles-day14; x = day 21.

**Figure 6:**
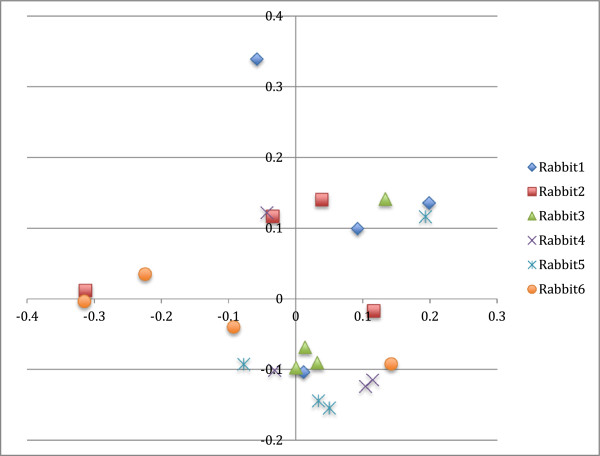
**Three dimensional principle coordinate analysis, highlighted by rabbit.** Legend: 5b: x: rabbit 1; open box rabbit 2, solid circle rabbit 3; solid triangle rabbit 4; open triangle, rabbit 5; open circle, rabbit 6.

Principle coordinate analysis results are presented in Figures 5 and 6. Using AMOVA, there was no difference between days using the Yue & Clayton index (*P* = 0.082), but there was a difference using the Jaccard index (*P* = 0.011), with significant differences between days 0 and 21 (*P* = 0.002) and days 14 and 21 (*P* = 0.030).

## Discussion

Rabbits enrolled in this study did not show any abnormal clinical signs, hematological values, postmortem evaluation, or physical changes of the hard feces throughout the observed study period, thus suggesting that the tested meloxicam dose can be considered safe for clinical use.

This study identified marked (but perhaps unsurprising) richness and diversity of the fecal microbiome that is incomparable with culture-dependent studies, as an average of over 2000 OTUs were identified. This and the corresponding diversity indices highlight the complexity of the rabbit fecal microbiota, even in rabbits housed under controlled conditions with little or no variation in diet and the need to use broad approaches such as this to understand this environment.

The predominance of Firmicutes was not particularly surprising, as this has been reported in other culture-independent studies of the rabbit [13] and can be found in other hindgut fermenters such as the horse [23,24]. It was interesting to observe that Ruminococcaceae and Lachnospiraceae were leading members of this phylum. Those two families are receiving increasing attention in various species as potentially key components of the ‘beneficial’ microbiota [23,25,26]. There is limited information about these bacteria in rabbits, although they were also reported to be dominant families in a study of 9-week old rabbits [27]. These may be critical components of the rabbit microbiome and groups to study further in terms of gut health and the loosely defined ‘intestinal dysbiosis’ that is often diagnosed in rabbits with gastrointestinal disease [28].

While the predominance of Firmicutes was not unexpected, the utter dominance of this phylum was remarkable. In studies of the fecal microbiome of most species, while one phylum may be most common, there are typically multiple phyla with relative abundances greater than 5% (or higher) [24,29-31]. Here, only one phylum beyond Firmicutes accounted for greater than 1% of sequences. That was Verrucomicrobia, which accounted for an average of 4.4% of sequences. Verrucomicrobia is a relatively newly described and poorly characterized phylum. It has been reported in similar relative abundances in other species, including horses [24,32,33], and has been shown to increase in abundance in mice fed type 2 resistant starch [34], perhaps indicating an important role in hindgut fermentation. *Akkermansia* (a member of this phylum) has been shown to be decreased in obese versus lean children and mice [35,36], to be associated with improved glucose homeostasis in metformin treated mice [37], to be associated with reversal of high fat diet induced metabolic disorders in mice [36] and to be reduced in humans with ulcerative colitis [38]. These suggest that this phylum may play an important role in gastrointestinal health in rabbits.

The number of sequences that were unclassified at the phylum level was large, although within the range that has been reported in other fecal microbiome studies and lower than that reported in earlier cloning-based studies of the rabbit cecal microbiota [10,39]. Even within sequences classified as members of the main phyla, Firmicutes and Verrucomicrobia, there were large percentages of sequences that were not identified to lower taxonomic orders. The relevance of these unclassified organisms is unclear. Unclassified sequences are not necessarily unknown phyla. Unclassified could indicate a sequence that does not have any closely related sequences in the reference database, but it could also indicate an inability of the analysis to assign a sequence to known phyla (or lower taxonomic levels) because of similarities in sequences or because of limitations in discriminatory power. Yet, unknown phyla could be present, particularly because of the paucity of knowledge regarding the fecal microbiota. The relevance of these in health and disease is completely unknown but given the high relative abundance, it must be assumed that this unclassified component of the microbiota could be of importance. This underscores our limited understanding of this complex polymicrobial ecosystem and the need for further study to define the microbiota and its individual constituents.

When assessing the microbial composition of these rabbits, there was surprisingly little clustering by individual, as is evident from the dendrograms and PCoA plot. With studies of other species, it is typically reported that there is less intra-individual variation than inter-individual variation [40,41], and if that was the case here, clustering by animal would have been expected. There are a few potential explanations. It is possible that there was enough of an effect of treatment to alter the microbiota of each rabbit, thereby disrupting intra-individual consistency, but that the alternation was not in a consistent or strong enough manner to result in a statistically significant effect of sampling day. Another more likely explanation might be the nature of this rabbit population, with similar genetics, housing and diet that resulted in a very homogenous microbiota between individuals. Study of variation in the microbiota of rabbits outside of a research colony would be enlightening. It is also possible that there is more inherent variation in the rabbit microbiota, since a study of rabbits reported similar intra- and inter-rabbit variation [15]. However, that study also involved a homogenously managed group of research rabbits so care must be taken when interpreting the degree of inter- and intra- individual variation from these studies.

Little impact of treatment was identified and there were inconsistent results between different tests. Differences were noted between days 0 and 21 using parsimony test and AMOVA, as well as days 14 and 21 using AMOVA. However, these were only when analysis was based on the Jaccard index. It is important to consider differences between these indices. The Yue & Clayton index is a measure of population dissimilarity based on both microbial presence and abundance, while the Jaccard index compares community membership without assessment of the relative abundance of different members of the community. Thus, differences in Jaccard but not Yue & Clayton indices suggest that changes in the community membership (addition or loss of members) occurred, but that this may have mainly involved low abundance members of community based on a lack of difference when relative abundance is considered. Other than the potential long-term microbiota adjustment to a new diet [42] the accumulated effect of meloxicam should also be considered, and in the absence of clinical or histologic abnormalities, only advanced molecular testing methods had the sensitivity to detect subtle changes in the rabbit’s GIT microbiota.

Limitations of the study must be considered. Since there was no untreated control group raised in parallel with these animals and they were all treated at the same time, it is possible that there was some unidentified factor such as a subtle change in diet or stress that accounted for the microbiota change. Additionally, it is known that the fecal microbiome of laboratory raised rabbits is different from rabbits raised under other conditions (e.g. farms, households) [42-44]. As with similar studies, there is always the question of how well the fecal microbiome represents the microbiome in more proximal sections of the gastrointestinal tract. That is an inherent limitation given the inability to easily and non-invasively obtain samples from different areas of the gastrointestinal tract over time. It has been reported that hard feces (which were studied here) are less representative of the cecal microbiota than soft feces [45] but conflicting data have also been reported [15,16]. These data should therefore be interpreted as a description and comparison of the hard fecal microbiota. Optimal approaches for studying the proximal intestinal tract in rabbits are not known. The sample size may also have limited statistical power; however, given the depth of data that are obtained and the similar sample sizes that have been used in other fecal microbiome studies, the sample size was deemed adequate to identify significant changes in the microbiota.

## Conclusions

The fecal microbiome of the rabbit is a complex and diverse polymicrobial ecosystem, composed of a wide range of both well-understood and completely unknown bacteria. While there were some noted differences over time, there was no clear and consistent impact of meloxicam treatment on the fecal microbiome. Given the importance of the intestinal microbiome in health and disease, particularly amongst hindgut fermenting species such as the rabbit, it is clear that there is inadequate understanding of the ‘normal’ microbiota, how it varies within and between rabbits, and how this complex polymicrobial ecosystem influences (and is influenced by) disease, nutrition, management and myriad other factors.

In the absence of adverse clinical, histologic and significant microbial changes observed in rabbits enrolled in this study, it is suggested that alteration of the hard fecal microbiota is not a significant adverse reaction expected with daily oral meloxicam administrated at the tested dose.

## Availability of supporting data

The data set supporting the results of this article are available at the MG-RAST metagenomics analysis server (project 8102, http://metagenomics.anl.gov).

## Endnotes

^a^Western Timothy Hay, Oxbow Animal Health, Murdock, Neb.

^b^Essentials – Young Rabbit, Oxbow Animal Health, Murdock, Neb.

^c^Vortech Pharmaceuticals, Dearborne, MO.

^d^Metacam 1.5 mg/ml oral suspension, Boehringer Ingelheim Vetmedica, St Joseph, MO.

^e^E.Z.N.A. Stool DNA Kit, Omega Bio-Tek Inc., Doraville, GA.

^f^Roche, Mississauga, ON, Canada.

^g^Invitrogen, San Diego, CA.

^h^Invitrogen, San Diego, CA.

^i^Beckman Coulter Inc, Mississauga, ON, Canada.

^j^Roche, Mississauga, ON, Canada.

^k^Illumina Inc, San Diego, CA.

## Competing interests

The authors declare that they have no competing interests.

## Authors’ contribution

DE participated in fecal collection, data generation and interpretation, and manuscript preparation. JSW participated in data analysis and interpretation, and manuscript preparation. Both authors read and approved the final manuscript.
